# Supplementary material for: Computational analysis of 3D biopolymeric porous scaffolds for the in vitro development of neural networks
Source: PLoS One. 2025 Nov 12;20(11):e0335432. doi: 10.1371/journal.pone.0335432 (PMC12611101; doi:10.1371/journal.pone.0335432)
Supplement: Attachment — Submitted filename: Response to Reviewers.docx [file pone.0335432.s002.docx]

Dear reviewers,

We would like to thank you for reading our manuscript and to provide precious comments. We have addressed all the observations and made the necessary revisions to the text.

Response to Reviewer 1

**• The integration of homogenization theory with nutrient transport modelling is a valuable approach. However, the manuscript lacks a clear articulation of its novelty in comparison to existing literature. The authors should explicitly state the methodological advancements and explain how this framework contributes to scaffold modelling in the context of neural tissue engineering.**

We appreciate the reviewer’s helpful comment. We have revised the manuscript to better emphasize the novelty of integrating homogenization theory with nutrient transport modeling, particularly in the context of neural tissue engineering. While homogenization has been occasionally applied in the field of tissue engineering, most studies have focused on mechanical property prediction rather than nutrient diffusion. To the best of our knowledge, very few studies apply homogenization to model diffusive transport in complex, heterogeneous scaffolds, and even fewer do so in relation to neural tissue constructs. By introducing a homogenized macroscopic framework, our work allows for a computationally efficient yet accurate description of nutrient transport, which would otherwise require high-resolution, resource-intensive microscale simulations.

We have clarified in the revised manuscript that the key methodological advancement lies in the application of homogenization to calculate effective diffusion tensors from a representative porous microstructure, enabling reliable prediction of oxygen gradients within 3D scaffolds seeded with neural cells. We believe that our work is a valuable contribution in the design of i*n vitro* models for neuroscience research.

• **Several paragraphs, particularly in the Introduction and Discussion, are dense and contain overlapping or tangential information. Reorganizing the content will help highlight the study's core contributions and findings. For instance, the Introduction paragraph beginning with “Another type of 3D culture is represented by porous scaffolds...” jumps between mechanical stiffness, hypoxia, and metabolic waste without clear transitions. Streamlining these sections will improve readability and focus**.

We thank the reviewer for this insightful feedback. We have carefully revised and reorganized the Introduction and Discussion sections to improve clarity and coherence. In particular, we simplified dense sections, removed repetitive or off-topic content, and made the transitions between ideas clearer.

• **Comparisons with experimental data, such as oxygen levels or cell viability, are mostly qualitative. Where possible, the authors should provide quantitative benchmarks or error analyses to support model validation. For example, the statement “The calculated diffusion time was 1.5 minutes...” is informative, but its relevance should be discussed in the context of experimental oxygen measurements or biological outcomes such as viability or proliferation**.

No direct measurements of oxygen and glucose consumption were performed; however, experimental analysis focused on astrocyte proliferation on chitosan films. The results demonstrated that a sigmoidal fitting accurately describes the cell growth pattern, supporting the computational model’s assumptions regarding the dynamics of astrocyte proliferation. This agreement between the experimental growth behavior and the modeled growth curve contributes to the validation of the study’s conclusions, despite the absence of direct metabolic measurements.

• **Key modelling assumptions such as treating neural cells as spheres and neglecting dendritic or axonal extensions are briefly mentioned but not critically evaluated. For example, in the Methods section: “...neural cells were considered as spheres with a 10 μm diameter, neglecting the developing dendrites and the axons...” The potential impact of this simplification on transport dynamics and cell behaviour predictions should be discussed**

Thank you for this valuable comment. We acknowledge that the assumption of modelling neural cells as perfect spheres, while neglecting dendritic and axonal extensions, is a simplification. This approach was chosen to reduce model complexity and computational cost, particularly in the early stages of development where neurite outgrowth is limited. However, we agree that this assumption may affect predictions related to local transport dynamics and intercellular interactions.

We have now included a critical discussion of this limitation in the Methods/Discussion section of the manuscript, emphasizing that:

- The spherical approximation is most appropriate for early neural progenitors before extensive differentiation
- The lack of neuritic extensions may underestimate spatial constraints, connectivity, and potential barriers to diffusion
- Future work could incorporate more realistic morphologies to improve the accuracy of behaviour and transport predictions.

• **Several figures (e.g., Figs 5, 6, and 7) contain rich and informative data but are not adequately explained in the Results section. Each figure should be contextualized with a clear description of what it illustrates and its implications. For instance, Fig 5D presents oxygen concentration along the scaffold's symmetry axis, but the biological relevance—such as thresholds for proliferation or hypoxia-induced apoptosis—is not discussed**

We thank the reviewer for this valuable suggestion. We have expanded the discussion in the Results section to provide a clearer interpretation of the data presented in Figures 5, 6, and 7. In particular, we have contextualized each figure by explaining its biological implications, including references to critical oxygen thresholds related to hypoxia-induced apoptosis. The revised text now highlights how oxygen concentration profiles relate to scaffold performance and cell viability under different conditions.

• **Some sentences are overly long or contain multiple ideas, which hinders comprehension. Additionally, there are minor grammatical and phrasing issues throughout the manuscript. For instance, the sentence “...was chosen as unit cell” should be revised to “was chosen as the unit cell**.”

We appreciate the reviewer’s observation. We have revised the manuscript to improve clarity and readability, shortening overly long sentences. In addition, we corrected minor grammatical and phrasing issues throughout the text.

Response to Reviewer 2

1. **What specific biopolymeric materials were used in the scaffolds, and what are their advantages for neural tissue engineering?**

For the realization of the scaffolds two different biopolymeric materials were used. The microbeads were realized using chitosan. The scaffold composed of fibrous films was fabricated by electrospinning polylactic acid (PLA) and subsequently functionalized with a chitosan coating through perfusion. Chitosan, obtained through the deacetylation of chitin, is a cationic polysaccharide recognized for its favorable characteristics, including biocompatibility, biodegradability, and antimicrobial and antifungal activity. In addition, it shares structural similarities with various extracellular glycosaminoglycans and exhibits comparable morphogenetic functions. Owing to its advantageous biological and physicochemical properties, chitosan is increasingly employed in neural cell culture. It has been demonstrated to support neuronal adhesion, growth, and functional maturation in 2D cultures derived from both primary neurons and iNeurons co-cultured with astrocytes. [*Di Lisa D, Muzzi L, Pepe S, Dellacasa E, Frega M, Fassio A, Martinoia S and Pastorino L 2022 On the way back from 3D to 2D: chitosan promotes adhesion and development of neuronal networks onto culture supports Carbohydr. Polym. 297 120049, Andolfi A, Arnaldi P, Lisa D D, Pepe S, Frega M, Fassio A, Lagazzo A, Martinoia S and Pastorino L 2023 A micropatterned thermoplasmonic substrate for neuromodulation of in vitro neuronal networks Acta Biomater. 158 281–91 , Grasselli, Sara, et al. "Chitosan suitability to adhesion, neuronal differentiation and maturation of hiPSCs compared to Matrigel coated substrates." Carbohydrate Polymer Technologies and Applications 9 (2025): 100686.*].

Polylactic acid (PLA), one of the most widely used biodegradable polyesters derived from renewable resources, has gained relevant attention as a biomaterial due to its properties, such as its low cost and low [immunogenicity](https://www.sciencedirect.com/topics/pharmacology-toxicology-and-pharmaceutical-science/immunogenicity). PLA fibers provide topographical cues that influence neuronal orientation and outgrowth, supporting the formation of functional synaptic connections. These properties make them particularly suitable for applications in neural tissue engineering. Moreover, PLA can be easily functionalized with biomolecules, such as chitosan. PLA films combined with the biopolymer chitosan have been shown to improve cell adhesion and promote the development of 2D neuronal networks [*Damonte, Giacomo, et al. "Development of functionalized poly (lactide) films with chitosan via SI-SARA ATRP as scaffolds for neuronal cell growth." International Journal of Biological Macromolecules 273 (2024): 13276*8.].

2. **How does the porosity of the scaffolds affect nutrient diffusion and cellular behaviour within the neural networks?**

The scaffolds porosity plays a critical role in regulating both nutrient diffusion and cellular behavior within neural networks. High porosity facilitates the efficient diffusion of oxygen, nutrients, and metabolic waste throughout the scaffold, which is essential for maintaining cell viability and promoting healthy neural growth. Moreover, an interconnected porous structure enables better cell infiltration and neurite extension, which supports the formation of more complex and functional neural networks. However, excessive porosity may compromise the mechanical stability of the scaffold, while low porosity can limit nutrient transport and hinder cell migration. Therefore, an optimal balance between porosity and structural integrity is crucial for supporting cell proliferation, differentiation, and synaptic connectivity in neural tissue engineering applications.

3. **What comparisons were made between the computational analysis results and experimental findings, and how do they validate the study's conclusions?**

No direct measurements of oxygen and glucose consumption were performed; however, experimental analysis focused on astrocyte proliferation on chitosan films. The results demonstrated that a sigmoidal fitting accurately describes the cell growth pattern, supporting the computational model’s assumptions regarding the dynamics of astrocyte proliferation. This agreement between the experimental growth behavior and the modeled growth curve contributes to the validation of the study’s conclusions, despite the absence of direct metabolic measurements.

4. **Are there any limitations in the computational analysis that could affect the scalability or applicability of the scaffolds in clinical settings?**

One limitation of this analysis in terms of clinical scalability is the model’s exclusive dependence on oxygen concentration to regulate cell proliferation, while neglecting other critical metabolic factors such as glucose availability. In a clinical context, nutrient dynamics are far more complex, and cell behavior is influenced by a variety of biochemical cues. Furthermore, assuming a sigmoidal proliferation response solely to oxygen may oversimplify the heterogeneous and dynamic environment found in vivo. This simplification could limit the model’s predictive accuracy when translated to larger, more complex tissue constructs or patient-specific conditions, thereby challenging its direct applicability in clinical scenarios. Another modelling assumption that may affect physiological relevance is the representation of neural cells as perfect spheres, omitting dendritic and axonal extensions. While this geometric simplification reduces computational complexity—particularly in early developmental stages where neurite outgrowth is limited—it may compromise the accuracy of predictions related to local transport dynamics and intercellular interactions. Such abstractions, though useful for model tractability, could limit the ability to capture key aspects of neural tissue organization and function.
